# Commentary: Merging of long-term memories in an insect

**DOI:** 10.3389/fpsyg.2015.00826

**Published:** 2015-06-16

**Authors:** Gema Martin-Ordas, Tom V. Smulders

**Affiliations:** Institute of Neuroscience, Centre for Behaviour and Evolution, Newcastle UniversityNewcastle, UK

**Keywords:** non-human animals, insects, episodic memory, false memories, memory conjunction errors

Imagine that you are asked to remember a list of words (e.g., “inside” and “consult”). At test you have to recognize which words were in the list. Now imagine that one of the words that you have to recognize is “insult.” Was “insult” in the list? If you respond “yes,” you are making a *conjunction error* (Underwood and Zimmerman, 1973; Reinitz et al., 1992; Kroll et al., 1996); that is, you incorrectly recognize a novel word (insult), which is made up of parts of two previously studied items (“in,” “sult”), as being part of the previously studied word list. This example illustrates that human episodic memory (memory for events, such as the event of learning a word list) is reconstructive and not an accurate representation of previously experienced events (Roediger, 1996; Tulving, 2005). When recalling an event, we often rely on our store of general knowledge to fill in the gaps, or we confuse information from different sources (Schacter, 2001). Thus, errors (e.g., conjunction errors) constitute the main evidence for reconstructive processes in remembering. Understanding episodic memory as an evolved capacity implies investigating other animals besides humans. Over the last two decades comparative psychologists have mainly studied whether non-human animals (henceforth animals) accurately remember what happened, where and when [Clayton and Dickinson, 1998; see Zentall et al. (2008) and Fortin et al. (2004) for other empirical approaches]. However, the reconstructive nature of episodic memory in animals has received much less attention, although some studies have addressed false memories in animals. For example, artificial memories have been induced by targeted neuronal activation in genetically engineered mice (Liu et al., 2012) and flies (Claridge-Chang et al., 2009); and brain damaged rats behave toward novel objects as if they were familiar (McTighe et al., 2010). However, no research has investigated the reconstructive nature of memory in intact animals, which is crucial to understand the normal functioning of their memory systems.

In a recent study published in Current Biology, Hunt and Chittka (2015) did just that: in a series of experiments they investigated whether bees make memory conjunction errors. For the purpose of our argument we will focus on the last experiment, which we consider to be the most convincing one. Authors trained bees to first find a reward (sugar solution) in artificial flowers with a black and white (b/w) pattern (a ring in one group, a grid in another group). Next bees from both groups were rewarded on plain blue flowers but not in the previously rewarded b/w ones. At test 24 h later, bees were presented with the two types of artificial flowers previously experienced and two new types: blue rings and blue grids. Note that whereas for one group the blue grid was the *conjunction stimulus* and the blue ring only shared one feature with the previously experienced stimuli (i.e., *feature stimulus*), for the other group the opposite was true. Bees preferred searching in blue flowers. However, they quickly developed a preference for the corresponding *conjunction stimulus* (e.g., blue ring) over the b/w and the *feature* stimuli (e.g., blue grid). This design illustrates that bees merge features of previously experienced flowers and rules out simple generalization, as an explanation for bees' responses.

But is this preference for the conjunction stimuli in the second half of the experiment evidence for a memory conjunction error? The outcome is different from typical memory conjunction errors in humans. Firstly, humans make memory conjunction errors after short retention intervals, while the bees did not (first experiment; not described above) (e.g., Reinitz et al., 1996; Jones et al., 2007). Secondly, independent of the materials used, humans consistently identify *conjunction* (new items created by combining features of two previously studied items) and *feature* (new items created by combining a new feature and a feature of a previously studied item) items as being “old” items more often than they do with totally *new* items, but less often than they do for actual *target* (i.e., old) items (Jones and Jacoby, 2001). An explanation based on familiarity accounts for such findings (e.g., Jones et al., 2007). Note that bees were not presented with a *new* item (i.e., stimuli integrated by two novel features) in any of the experiments, and they preferred the *conjunction* stimulus (e.g., blue ring pattern for those subjects who experienced “ring” in the first part of the training) to *feature* (e.g., blue grid) and original stimuli (i.e., b/w ring pattern)—as mentioned above this preference only emerged during the second half of the test.

We think that the training procedure might have caused these differences. During the differential reinforcement phase bees learned which flowers contained reward and also which ones did not. However, the change (increase) in associative strength of the positively-reinforced flowers will be greater than the change (decrease) in associative strength in the non-reinforced flowers, because the bees visit more rewarded flowers than non-rewarded flowers (training data in Supplementary Materials). Therefore, after the reversal learning, there is still some positive association left with the firstly-trained flowers, albeit less than with the last-trained flowers (see Menzel, 1969). If we then assume that the associative strength is distributed between the two features of each flower (pattern: ring vs. disk; and color: black vs. blue), then the conjunction stimulus should have an associative strength somewhere between the two original stimuli. With the right combination of associative strengths assigned to the different features (which may have different salience), the results should come out as observed, with a preference for the last-rewarded type (blue), followed by the *conjunction* stimulus, and an initial avoidance of the *feature* stimulus and the last-non-rewarded type. A loss of associative strength over time (especially if that drop is steeper for the most recently-rewarded features) would explain why the pattern only arose after 24 h, but not on immediate testing.

Does the fact that we can explain Hunt and Chittka's (2015) results using associative learning mean that bees do not make memory conjunction errors? Not necessarily. There is no a priori reason to prefer an associative learning explanation to a more cognitive one. Both the associative learning and familiarity accounts, for example, would predict that training the bees such that both stimuli were rewarded simultaneously (or interleaved) should result in preference for both *target* stimuli, but might result in preference of *conjunctive* stimuli over *feature* stimuli and *new* stimuli. Finding conditions that distinguish between the two explanations might be difficult, as both could be true at the same time but at different levels of analysis.

Whether or not humans and bees retrieve features in the same manner or not, the approach of looking for similarities between the memory systems of humans and other animals by investigating “false memories” is an exciting one. We look forward to a new crop of papers exploiting this novel direction in comparative cognition.

## Conflict of interest statement

The authors declare that the research was conducted in the absence of any commercial or financial relationships that could be construed as a potential conflict of interest.
